# Active involvement of patients and relatives improves subjective adherence to hygienic measures, especially selfreported hand hygiene: Results of the AHOI pilot study.

**DOI:** 10.1186/s13756-019-0648-6

**Published:** 2019-12-12

**Authors:** Tillmann Görig, Kathleen Dittmann, Axel Kramer, Claus-Dieter Heidecke, Stephan Diedrich, Nils-Olaf Hübner

**Affiliations:** 1 Central Unit for Infection Prevention and Control, Universitätsmedizin Greifswald, Walther-Rathenau-Str. 49A, Greifswald, 17475 Germany; 2 Institute of Hygiene and Environmental Medicine, Universitätsmedizin Greifswald, Greifswald, Germany; 3 Polyclinic and Clinic for General Surgery, Visceral, Thoracic and Vascular Surgery, Universitätsmedizin Greifswald, Greifswald, Germany; 4 Medical Chairman of Universitätsmedizin Greifswald, Greifswald, Germany

**Keywords:** Prevention and control, Cross infection, Disease transmission, Hand hygiene, Health education

## Abstract

**Background:**

The prevention of nosocomial infections requires participation from the patients themselves. In the past, however, patients have been apprehensive to point out hygiene-relevant behaviour to the personnel.

In the project AHOI, the possibilities of active patient involvement in infection prevention are identified, tested and realized. The goal is a prevention strategy based upon three dimensions: “adherence”, “empowerment” and “acceptance”. “AHOI” stands for the “**A**ctivation of patients, persons in need of care and care givers for a **H**ygiene-conscious participati**O**n in **I**nfection control”. Results from the AHOI pilot study on the implementation of a multimodal intervention bundle are reported.

**Methods:**

In 2017, a two-stage patient survey was conducted on two surgical wards for 14 weeks. In addition to the intervention bundle, acceptance, adherence and empowerment regarding individual hygiene behaviour and perception were evaluated. The bundle included an AHOI-welcome-box with an informational and entertaining brochure and supportive incentives. Furthermore, multiple visual materials like video presentations for patients’ bedside TV, posters and visual reminders in the patients’ bedrooms and sanitary facilities were installed.

**Results:**

179 respondents were surveyed at admission, 139 at discharge and 133 at both time points. Almost all respondents wanted to contribute to infection control. The AHOI project was well accepted by patients. Two-thirds wanted to be more involved. More than a third expected a negative response from staff after pointing out hygiene deficiencies. Four respondents observed a deficiency in hygiene with healthcare personnel and reported a very positive reaction once this was communicated to the personnel. More than four-fifths of the respondents felt well integrated and adequately informed post intervention. The feeling of active involvement correlated significantly with subjective participation and adherence to hygienic measures, especially self-reported hand disinfection.

**Conclusion:**

The results demonstrated that the required inclusion of patients in infection control is possible with AHOI. Active involvement of patients and relatives is associated with improvements in adherence to infection prevention measures.

## Key points

- Patients expressed interest in being involved with infection control measures
- To date, patients had concerns to point out hygiene-relevant behaviour to the personnel
- Patients are open to hygiene - feedback from personnel
- Informational materials support hygiene-related emancipation and compliance in a team sensitized to patient involvement
- Patient education leads to improved hygiene behaviour in dialogue with the treatment team

## Introduction

Avoidance of hospital-acquired infections has become an integral part of patient safety [1]. Since creation of the World Health Organization (WHO) resolution “Quality of care: patient safety” at the 55th World Health Assembly in 2002, infection control and prevention (ICP) has also become a global focus [2]. Consequently the World Alliance for Safer Health Care was founded in 2004 followed by the “SAVE LIVES: Clean your Hands” campaign in 2009 [3, 4]. In 2007, this topic was adopted as a European public health issue by the European Commission in their White Paper “Together for Health: A Strategic Approach for the EU 2008-2013”. Subsequently, the Council of the European Union published their recommendation on patient safety, including the prevention and control of healthcare associated infections [5, 6]. It had become overall recognized that the participation of patients could represent one of the most obvious advantages in ICP and patient safety.

The German Commission for Hospital Hygiene and Infection Prevention (KRINKO) at the Robert Koch Institute (RKI) has increasingly provided recommendations for the involvement of patients in infection prevention. The German Alliance for Patient Safety (APS) has also demanded a stronger integration of patients into the treatment security. Kramer et al. were among the first in 2016 to include these general recommendations with concrete recommendations for action, e.g. on the behaviour in the patient and sanitary area [7].

The following is a presentation of the results from the “AHOI – patient on board” pilot study.

The letter word “AHOI” stands for the “**A**ctivation of patients, persons in need of care and care givers for a **H**ygiene-conscious participati**O**n in **I**nfection control”. It is an interdisciplinary and cooperative project of the Institute of Hygiene and Environmental Medicine, the Polyclinic and Clinic for General Surgery, Visceral, Thoracic and Vascular Surgery of the Universitätsmedizin Greifswald (University Hospital) and the Chair of General Business Administration and Health Care Management of the University of Greifswald.

Main goals of the project were the development and testing of a prevention strategy, consisting of three general dimensions:

Adherence (patients and visitors should be aware of hygiene standards and adhere to them),

Empowerment (patients and visitors should consciously perceive the hygienic behaviour of the personnel and should be able to address noticeable problems/abnormalities) and Acceptance (the personnel should convey the feeling to the patients and visitors, that they are at the same level in terms of ICP and patient safety) [8–10].

For this purpose, a multimodal intervention bundle was developed and implemented on two surgical wards for a pilot study. The here reported pilot study was conducted to test the feasibility of an implementation of the intervention bundle and used a survey to evaluate the developed material. The survey was also conducted to gain initial insight into the integration of patients, their relatives and visitors into infection control in a hospital environment. The patients were questioned upon admission as well as discharge about their wishes, expectations and perceived integration into the infection prevention program. They were also asked to evaluate the AHOI-informational materials which had been provided and the expected and perceived behaviour of healthcare personnel.

## Methods

### Study design

The study was designed as a feasibility study with a cross-sectional design. It’s primary goal was to question the implementation and acceptance of the developed AHOI intervention as well as to possibly follow behavioural changes in patients and healthcare personnel.

The study was approved by the medical ethics commission, Universitätsmedizin Greifswald (BB 087/16b) and is reported following the CONSORT guidelines [11].

### Participants and interventions

The developed AHOI intervention was carried out on two wards of the Polyclinic and Clinic for General Surgery, Visceral, Thoracic and Vascular Surgery for a time period of 14 weeks (26 January – 03 May 2017). Participants were surgical and medical patients. Patients over 18 years were eligible to participate. Exclusion criterion were inability to realize the AHOI concept, e.g. due to lack of German language knowledge, mental confusion or advanced dementia. The participation was voluntary and pseudonymized.

**Fig. 1:**
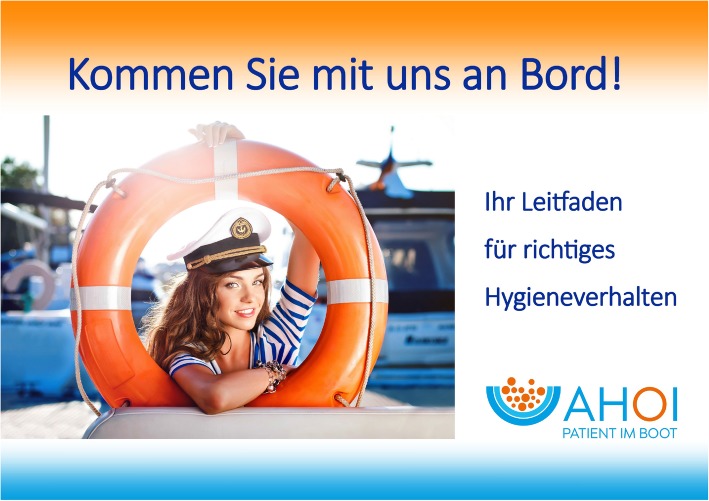
AHOI-Brochure Cover

A multimodal package of informational and motivational material was implemented, including e.g. posters, brochures and video presentations for patients. An essential part was the “AHOI-welcome-box” that was handed out to patients upon admission (see Additional file 1). This contained not only supportive incentives but also the AHOI brochure which is a motivational hygiene guide with entertaining images (Fig. 1). The brochure contains information on different aspects of infection prevention behaviour for patients and relatives in the hospital and at home. In particular, instructions for hand hygiene, food hygiene, bathroom hygiene, and hygiene behaviour as a patient as well as information on antibiotic intake are given. Other topics include signs for infections, behaviour under contact precautions, attention to correct standard precaution of the personnel as well as communication tips to be able to address abnormalities politely but directly. The incentives consisted of disinfectants, handkerchiefs, a pen, a notepad, a bag, a nail case and a hygiene-relevant puzzle booklet. Visual reminder aids for a correct ICP behaviour were installed in the patient rooms and sanitary facilities as well as in the corridors. The two videos (“Mention It!” and “Stay Clean – disinfect your hands!”) were played as a continuous loop on screens in the entrance hall and on a separate channel in the patient’s bedside TV. The treatment team of both wards received a six-hour psychological curriculum. The curriculum conveys the AHOI approach and its background. The healthcare personnel should get introduced to AHOI and learn to accept patients who are more involved in infection prevention. Key aspects of the interdisciplinary train-the-trainer-teaching were communication tools and skills and role-playing-elements. The healthcare personnel were surveyed with an independent questionnaire on their expectations, observations and perceptions (data not shown).

### Outcomes and data collection

Primary research questions were the feasibility to implement the developed AHOI instruments and their acceptance by the involved persons. This included the patients’ evaluation of the AHOI materials and their perception of changes in behaviour, adherence and empowerment of themselves, their relatives and the personnel.

Data collection was questionnaire based, mainly with closed questions which were nominally or ordinally scaled (e.g. “yes – no”; or as a 10-point scale: “negative – positive”). The patient’s age was recorded with a 6-point scale: 1 = 18–20 years, 2 = 21–30 years, 3 = 31–45 years, 4 = 46–60 years, 5 = 61–70 years, 6= > 70 years. Educational history was recorded with the categories: no graduation, school graduation, vocational training graduation, or university degree. Half-open and open questions were integrated to enable specific answers. The amount of questionnaires and the return rate were recorded. To avoid priming of the respondents, the questionnaire was split into two parts [12]. An ID realized matching of both parts. Every respondent received the questionnaires and two blank envelopes for the anonymous return within the AHOI box. They were requested to answer the first part directly at the beginning without looking at the rest of the content. The second part had to be filled out at the last day of their stay. An information sheet in the box invited the respondents to participate voluntarily and anonymously. The return of the questionnaires in closed envelopes was therefore viewed as informed consent. Study nurses were responsible for distribution of the AHOI boxes whereby the respondents were informed about the goal of the study and motivated to participate properly according to the procedure. Overall 310 boxes with 620 questionnaires were given out.

The question catalogue covered five main categories:

1. Role-understanding, perception of integration into the treatment security and wish for integration
2. Expectations and observations in interaction with the personnel (Empowerment)
3. Acceptance of the changed roles of the healthcare personnel through AHOI from the perspective of patients
4. Knowledge and implementation of hygiene standards (Adherence)
5. Evaluation of information materials

### Data processing and data analysis

The collected data was entered in an active PDF-format (Adobe Acrobat XI) by two independent research assistants and subsequently exported to SPSS. IBM SPSS Statistics 22 (Version 22.0; IBM Corporation, Armonk, NY, USA) was then used for data comparison, correction and statistical analysis. All presented percentages were rounded to the first place after the decimal point. Beside descriptive statistics, inferential methods like correlation tests of Spearman Rho and Pearson’s R etc. were applied and reported when significant and decisive results were found.

Informational data aimed at a direct comparison between the two time points, was evaluated only from questionnaires where both questionnaire sections were available. For questions appearing in only one section, full individual sample size was reported.

## Results

### Distribution and return rate

From the 310, two-part questionnaires distributed upon admission or discharge, 179 (57.7%) and 139 (44.8%) respectively were filled out and returned to the study centre. The total return rate was 51.3%. Precisely, 133 (42.9%) respondents returned both the admission and a discharge questionnaire section (Table 1).

**Table 1:** Distributed and returned admission and discharge questionnaire sections with return rate

	Admission section	Discharge section
Distributed	310 (100%)	310 (100%)
Usable return	179/310 (57.7%)	139/310 (44.8%)
Total return	318/620 (51.3%)	
Matching Respondents	133/310 (42.9%)	

Legend: Matching Respondents = only the respondents who returned both sections for admission and discharge

### Descriptive statistics

The proportion of female and male respondents was approximately the same (female/ male: 46.6%/ 53.4%, Table 2). Nine respondents made no indication of gender. The median value of age was 46–60 years. The majority reported vocational training as their highest professional qualification when asked for educational standing (Table 2). The gender-, age distribution as well as educational standing of the respondents who had answered both sections (matching respondents) are shown in Table 2.

**Table 2:** Distribution of sex, age and educational standing of all respondents and of matching respondents

	All Respondents		Matching Respondents	
	%	Frequency	%	Frequency
Sex				
Female	46.6	82/176	51.1	67/131
Male	53.4	94/176	48.9	64/131
Age				
18–20 years	1.1	2/178	1.5	2/133
21–30 years	6.7	12/178	8.3	11/133
31–45 years	15.2	27/178	18	24/133
46–60 years	28.7	51/178	26.3	35/133
61–70 years	27	48/178	26.3	35/133
over 70 years	21.3	38/178	19.5	26/133
Educational standing				
No graduation	2.1	3/141	2.8	3/107
School graduation	13.5	19/141	11.2	12/107
Vocational training graduation	55.3	78/141	51.4	55/107
University degree	29.1	41/141	34.6	37/107
Length of stay	Mean ± standard deviation		Mean ± standard deviation	
Mean length of stay	6.4 ± 5.5	126/139	6.3 ± 5.4	121/133

Legend: All Respondents = all respondents who returned a questionnaire section regardless of whether they returned only the section for admission or discharge. Matching Respondents = only the respondents who returned both sections for admission and discharge

### Role-understanding, perception of integration and wish for integration

Almost all respondents stated upon admission that they wanted to contribute to infection protection through their own behaviour (“fully agree” or “rather agree”: 97.6%). More than 2/3 of the respondents also expressed the wish to be more involved in infection control (“yes”: 67.4%).

**Fig. 2:**
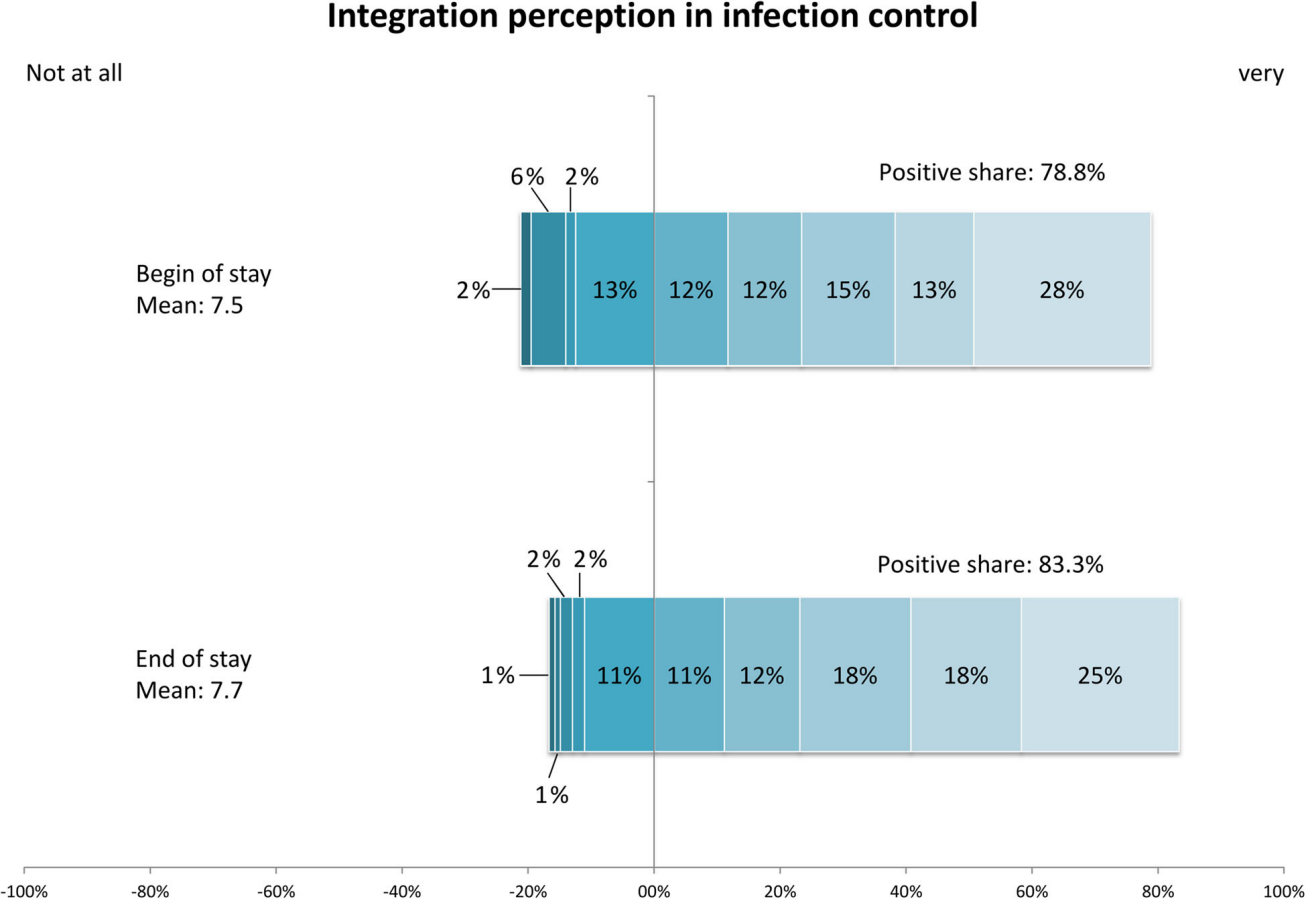
Integration perception in infection control of patients at the beginning and at the end of stay. 10-point scale, admission *n* = 128, discharge *n* = 108, Information is rounded to the left comma digit

Upon admission, actual involvement in infection control was rated as positive by three-quarter of the respondents (78.9%). Upon discharge, the assessment of actual involvement in infection control had noticeably risen to 83.3% (Fig. 2).

### Empowerment: expectations and observations in interaction with the personnel

#### Expectations

**Fig. 3:**
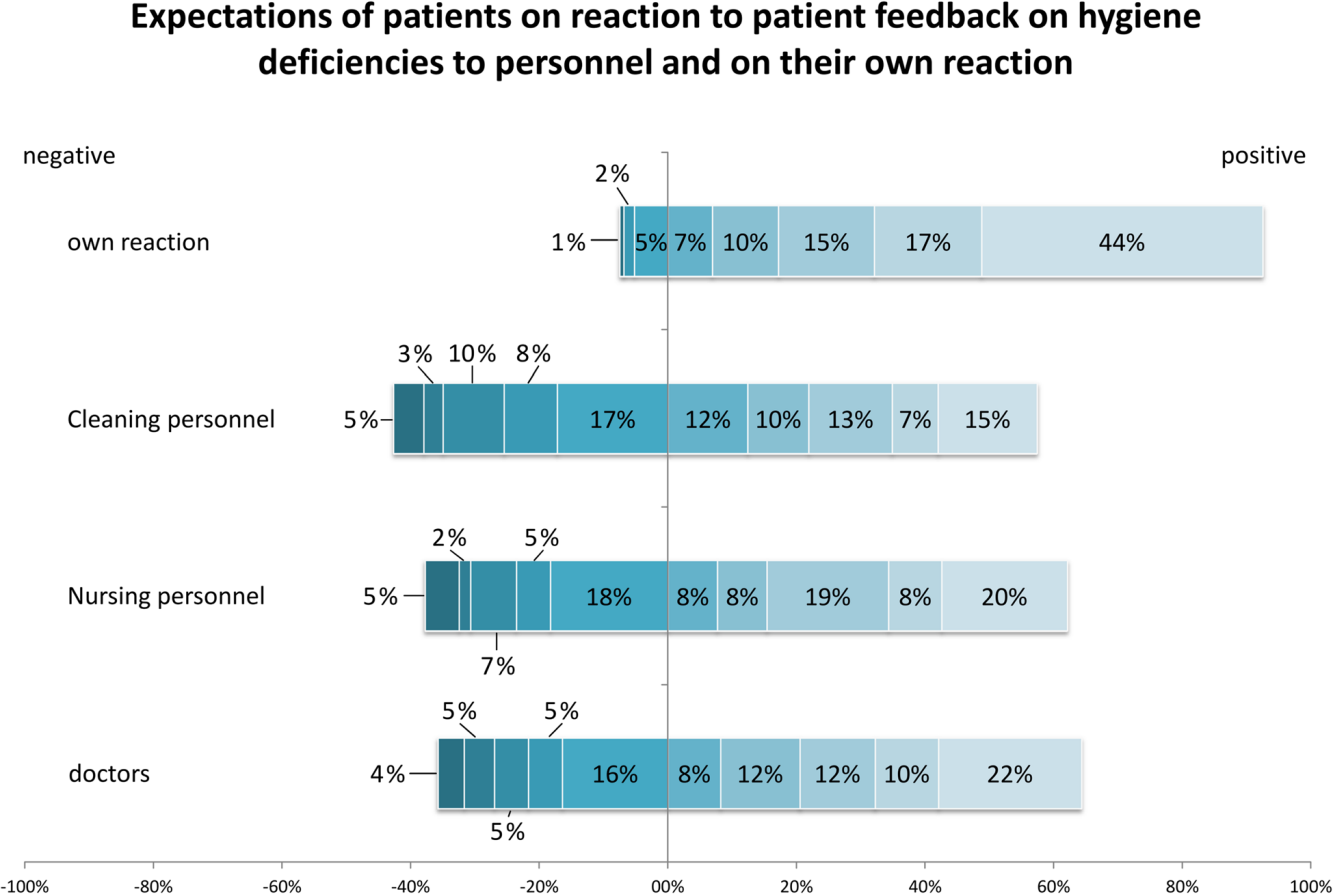
Expectations of patients on reaction to patient feedback on hygiene deficiencies to personnel and on their own reaction. Doctors *n* = 171, nursing personnel *n* = 169, own reaction *n* = 174, cleaning personnel n = 169

Patient expectation of the personnel’s reaction to the reporting of hygiene deficiencies was questioned for each occupational group of the personnel (doctors, nursing personnel, cleaning personnel) (Fig. 3).

Upon admission, 35.7% of respondents expected a negative response (5 and worse on a 10-step scale) from physicians responding to a feedback of hygiene deficiencies. Expectations for the reaction of nurses were comparably negative (37.9%). Respondents expected the most negative response from the cleaning personnel (42.6%). Interestingly, the respondents estimated their own reaction to direct feedback on hygiene behaviour far more positive (92.5%).

#### Observations

*All Respondents*

Upon admission, 31.6% of respondents reported that they had observed hygiene deficiencies during past hospital and doctor visits. At the time of discharge, 15.9% of respondents reported having observed hygiene deficiencies during their current stay.

Of the 56 respondents upon admission and 20 respondents upon discharge who reported a lack of hygiene during a past or current stay, 39 (69.6%) and 16 (80%) respectively answered the question of whether they communicated this to the personnel. Of these, 15 (38.5%) upon admission and 4 (25%) upon discharge stated that they had shared their observations with the personnel.

*Matching Respondents*

Of the respondents who completed both sections of the questionnaire, 31.8% reported deficiencies observed in previous treatments. Upon discharge, 16.7% of these respondents reported having observed hygiene deficiencies during their stay. Of those who reported a lack of hygiene, 33.3% indicated at admission and 25% upon discharge to have reported their observations to the personnel.

At the end of their hospital stay, only a few respondents reported experience with personnel responses or feedback on their own hygiene behaviour. The four respondents, who observed and reported a deficiency, experienced a very positive reaction of the personnel (mean 9.25 on a negative-positive scale of 1 to 10).

Upon discharge, five respondents (4.2%) reported that they had been approached by personnel about hygiene issues or better hygiene behaviour during their current hospital stay. Four of these respondents indicated that they personally reacted very positively to the feedback (mean 9.25 on a negative-positive scale of 1 to 10).

### Acceptance

The personnel’s acceptance of the AHOI-altered role of the patients was recorded with several items. A directly measurable indicator was the distribution of the AHOI intervention materials. An indirect indicator was the active addressing of AHOI during the intake interview which was conducted at a different time. The boxes were distributed to all questioned patients (100%). However, only 64.9% reported that they had been actively approached on the topic of AHOI in the intake interview.

### Adherence

Upon discharge, almost all respondents stated that they felt they had been sufficiently informed about the personal ICP behaviour in the hospital (“yes”: 96.2%). Similarly, the majority reported knowing what they could look out for concerning the personnel (85.1%).

The reported high level of awareness and the feeling of being involved in infection control (83.3%) also found expression in the responses to one’s own behaviour. Of the 111 respondents, 94.6% said they had participated in the prevention of infection (in the direction of the scale-point: “very much”). Compliance to hand disinfection through use of the disinfectant dispensers for the “5 moments of hand disinfection”, was reported by 58.2% of the respondents to be performed “almost always” and by 36.6% to be performed “frequently” (see Additional file 2).

### Integration and self-reported compliance

Self-reported hand disinfection compliance and self-rated participation (“How often have you used the disinfectant dispensers? - According to the 5 moments for hand disinfection” and “In your opinion, how much did you participate in preventing infection?”) clearly correlated with the involvement perception in infection prevention (“How well involved into infection control did you feel during your stay”). Self-rated participation aims at the subjective assessment of the patients as how active they were themselves. Involvement perception aims at the assessment of the patients as how well did they feel integrated in the infection control system. Respondents, from whom both sections of the questionnaire were available, showed a clear, statistically significant correlation between involvement perception and self-rated participation (r_s_^1^= 0.617, *n* = 103, *p* < 0.0001).

The self-reported use of the disinfectant dispenser for the “5 moments of hand disinfection” also correlated statistically significant with a medium effect with the involvement perception (r_s_ = 0.376, *n* = 106, p < 0.0001).

The equipment with hand disinfectant dispensers in patient areas and their accessibility was evaluated positively. Most particularly highly rated were the disinfectant dispensers located directly within the patient room units and sanitary areas (99.3%).

### Evaluation of intervention material

The AHOI-box as a whole was rated “useful and helpful” by 98% of the respondents. The AHOI brochure as the core element of the box left a good impression on 73.8% of the patients and a “medium” impression on the remaining 26.2% (see Additional file 3).

The information videos were rated more critically. 65.6% gave a “good” rating on the overall impression of the information videos.

The reminders (posters, etc.) were rated more positively in comparison. 78.2% of respondents stated that the visual materials helped to comply with the hygiene measures.

## Discussion

Nosocomial infections and their transmission (especially multi-resistant pathogens) are among the greatest concerns associated with inpatient hospitalisation [13].

The active involvement of patients in infection prevention has been taken into serious consideration in Germany. Not only by the APS, but also within recommendations of the KRINKO which form the basis of infection prevention in German hospitals [14–17].

With “AHOI - patient on board”, a multimodal concept was developed for the first time in Germany in order to systematically realize these requirements. The presented pilot study served to examine the feasibility of the concept. The introduction of AHOI materials was accompanied by questionnaire-based surveys to assess the preconditions, feasibility and effects of the intervention.

The results revealed that the predominant majority of respondents wished for a greater involvement in infection control and agreed to a more active role. The AHOI intervention was shown to be suitable for fulfilling this desire. The reported strong feeling of being actively involved in infection prevention during hospitalisation significantly correlated with a high level of adherence to hygienic measures. Specifically, it could be shown that self-reported compliance with hand disinfection correlated significantly with the feeling of integration. This is in line with other studies [18–21] and shows that KRINKO’s and APS’s demand for advocating hand disinfection for patients in AHOI is viable.

The AHOI materials used were generally rated positively. The AHOI boxes performed especially well. The study, however, also made clear the hurdles and possibilities of patient integration. More than a third of the respondents expected a negative reaction from healthcare personnel when reporting hygiene deficiencies. This negative expectation was in marked contrast to the positive assessment of the respondents’ own reaction to feedback of their own hygiene deficiencies. That could indicate a communicative misunderstanding. Most patients may not see any problems in open communication pertaining to hygiene deficiencies, but rather welcome a constructive error feedback. At the same time, however, they do not expect the same level of acceptance from the personnel and fear a negative side-effect.

One goal of AHOI is to contribute to a constructive culture of communication through employee education and empowerment of the patients. The positive reports on the actual reactions of the healthcare personnel show that a positive safety culture is possible.

However, the clear difference between perceived and addressed hygiene deficiencies shows that prolonged intervention is required to overcome the communication barrier.

These findings coincide with the results of other authors [18, 19, 22–25]. Furthermore, an additional report will be published concerning the AHOI-survey of the participating healthcare professionals.

### Limitations

Our study has limitations that need to be considered into the assessment. It is a monocentric feasibility study under controlled conditions. Due to the design, sample size and the duration of the study, only limited statements on effects are possible.

In particular, statements to the effectiveness of the measures are only indicative. Since some statements are only possible based upon sequence of events (e.g. in response to reported hygiene deficiencies that require the occurrence, perception and expression of the same), in some instances sample sizes have emerged which are more likely to be viewed as case-by-case reports.

The respondents were asked to assess their own active hand disinfection behaviour and their personnel perception of patient integration into infection control on the AHOI wards. Admittedly, for the time being we cannot rule out the possibility that both items are measuring in some way the same or analogous aspect of hygiene behaviour. Suppose the respondents are assessing the self-reported compliance analogous to the integration feeling because of a social desirability bias. That could be interpreted positively because that means the respondents were aware of the intention of the intervention and therefore reported a quite positive compliance which would be a desired result.

Nevertheless, we consider the basic statements of the study to be sound. The high response rate, the positive evaluation of the materials and the same directional changes in adherence, empowerment and acceptance items demonstrate the feasibility and potential of AHOI to systematically advance the still novel topic of patient integrated hygiene in Germany. Further efforts are needed to prove the effectiveness of the measures and to find ways to overcome the identified barriers. Another expanded AHOI-study is currently being conducted to address the deficits of the presented study.

## Conclusion

The results indicate that the recommendations of the KRINKO and the APS are in line with the interests of the patients and that an improved involvement in the prevention of infection meets the wishes of patients. The reported strong feeling of being involved in infection prevention by AHOI clearly correlated with a high level of adherence to hygienic measures, especially hand hygiene. The AHOI concept has proved to be feasible and is a way to implement KRINKO’s recommendations.

## Supplementary information

**Additional file 1: Figure S1**. Content of AHOI-Box.

**Additional file 2: Figure S2.** Use of disinfectant dispenser by patients, *n* = 134.

**Additional file 3: Figure S3.** Evaluation of AHOI-films overall and of the brochure, films *n* = 209, brochure *n* = 126.

## Data Availability

The datasets generated and/or analysed during the current study are not publicly available due to protective measures because of the limited study duration and sample size but are available from the corresponding author on reasonable request.

## Abbreviations

AHOI: Activation of patients, persons in need of care and care givers for a hygiene-conscious participation in infection control, AHOI project, Institute of Hygiene and Environmental Medicine
APS: Alliance for Patient Safety, Germany
KRINKO: Commission for Hospital Hygiene and Infection Prevention, Germany
RKI: Robert Koch Institute, Germany
WHO: World Health Organization

## Footnotes

1 Spearman’s rho correlation coefficient.
