# Supplementary figures and images for: Active involvement of patients and relatives improves subjective adherence to hygienic measures, especially selfreported hand hygiene: Results of the AHOI pilot study
Source: Antimicrob Resist Infect Control. 2019 Dec 12;8:201. doi: 10.1186/s13756-019-0648-6 (PMC6909614; doi:10.1186/s13756-019-0648-6)

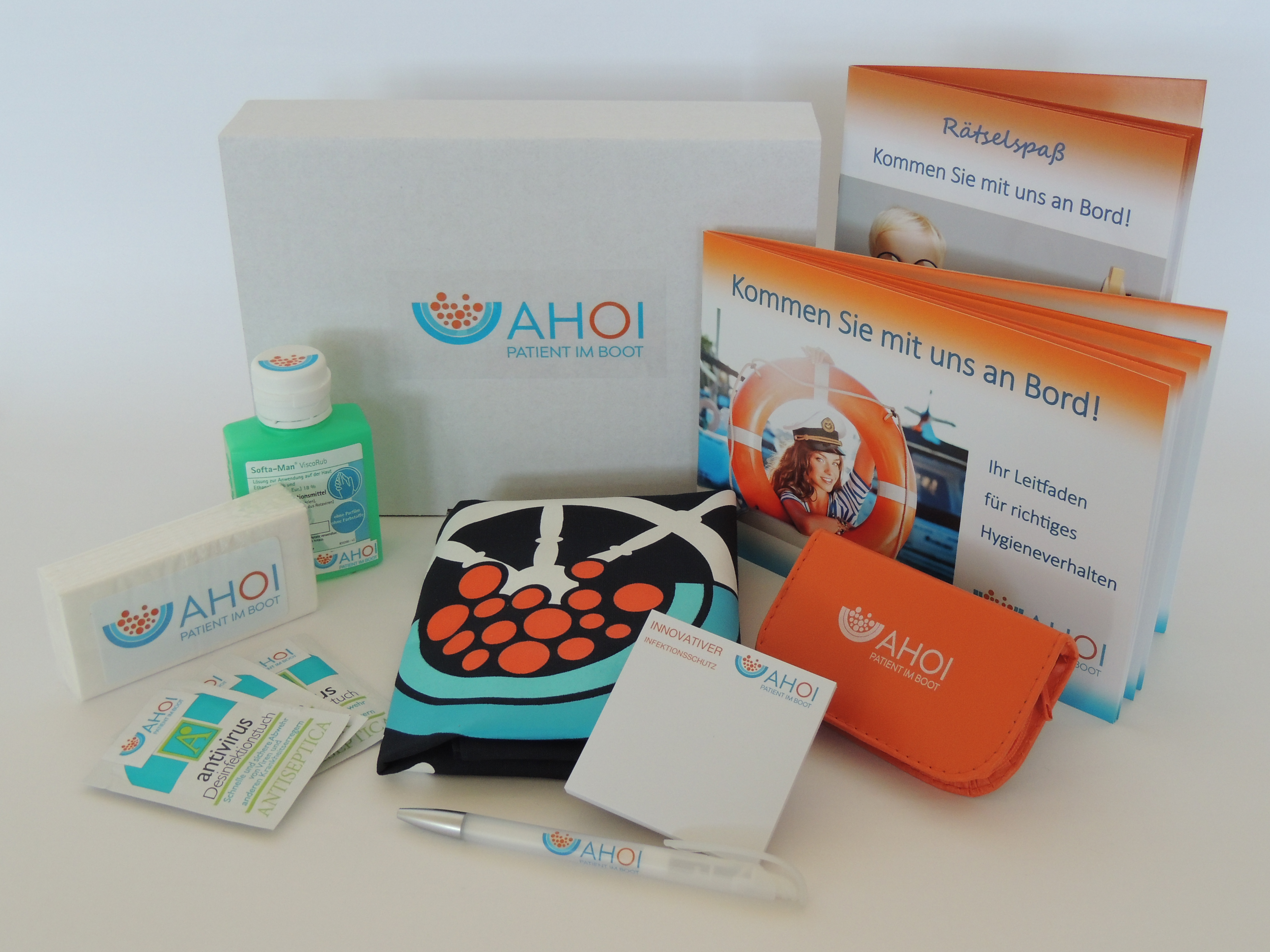

Supplement: Supplementary file 1 — Additional file 1: Figure S1. Content of AHOI-Box. [file 13756_2019_648_MOESM1_ESM.jpg]
