# Supplementary material for: Active involvement of patients and relatives improves subjective adherence to hygienic measures, especially selfreported hand hygiene: Results of the AHOI pilot study
Source: Antimicrob Resist Infect Control. 2019 Dec 12;8:201. doi: 10.1186/s13756-019-0648-6 (PMC6909614; doi:10.1186/s13756-019-0648-6)
Supplement: Supplementary file 2 — Additional file 2: Figure S2. Use of disinfectant dispenser by patients, n = 134. [file 13756_2019_648_MOESM2_ESM.pdf]

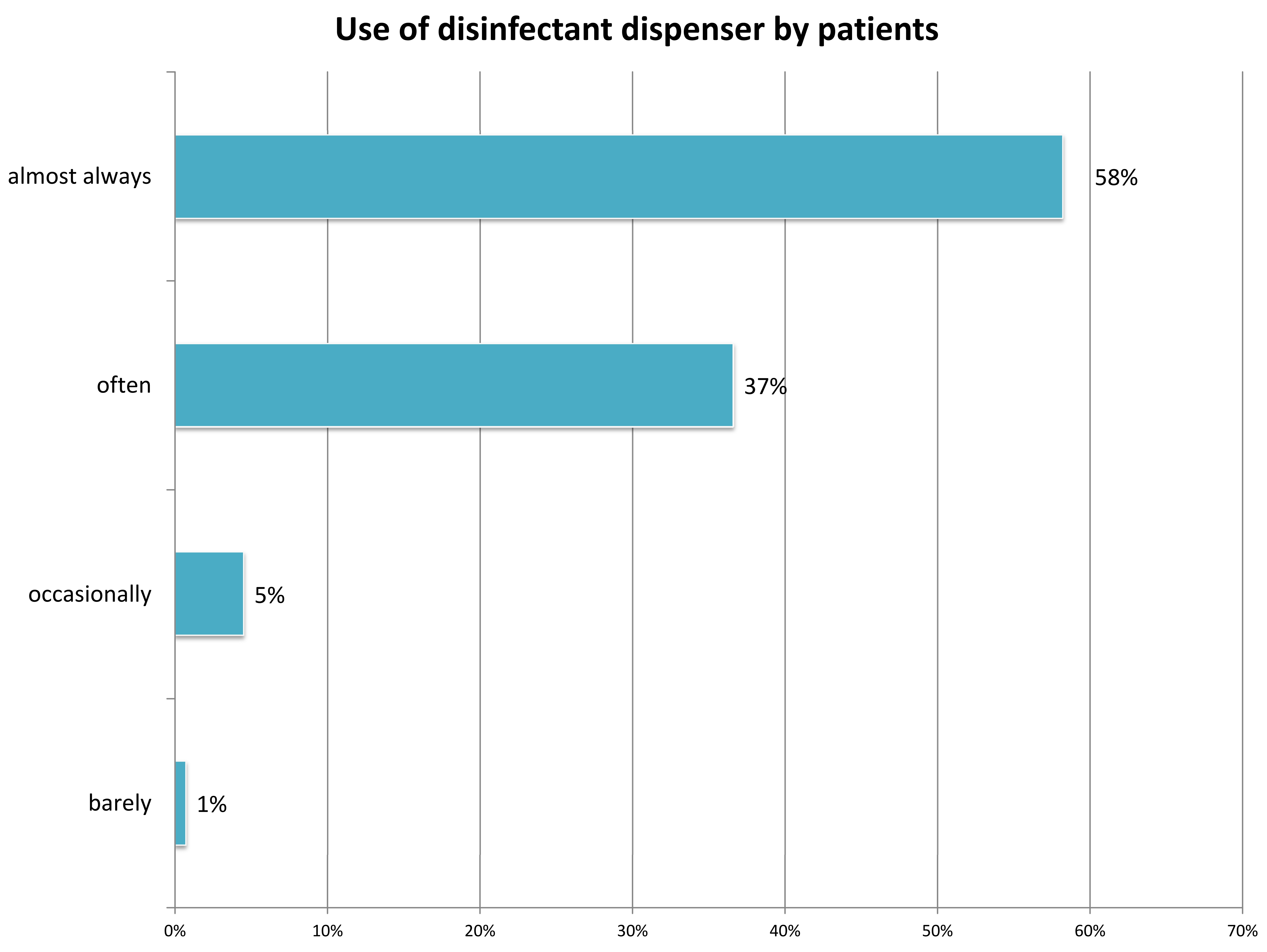
Use of disinfectant dispenser by patients
almost always
58%
often
37%
occasionally
5%
barely
1%
0%
10%
20%
30%
40%
50%
60%
70%
