# Supplementary material for: Active involvement of patients and relatives improves subjective adherence to hygienic measures, especially selfreported hand hygiene: Results of the AHOI pilot study
Source: Antimicrob Resist Infect Control. 2019 Dec 12;8:201. doi: 10.1186/s13756-019-0648-6 (PMC6909614; doi:10.1186/s13756-019-0648-6)

# Evaluation of AHOI materials

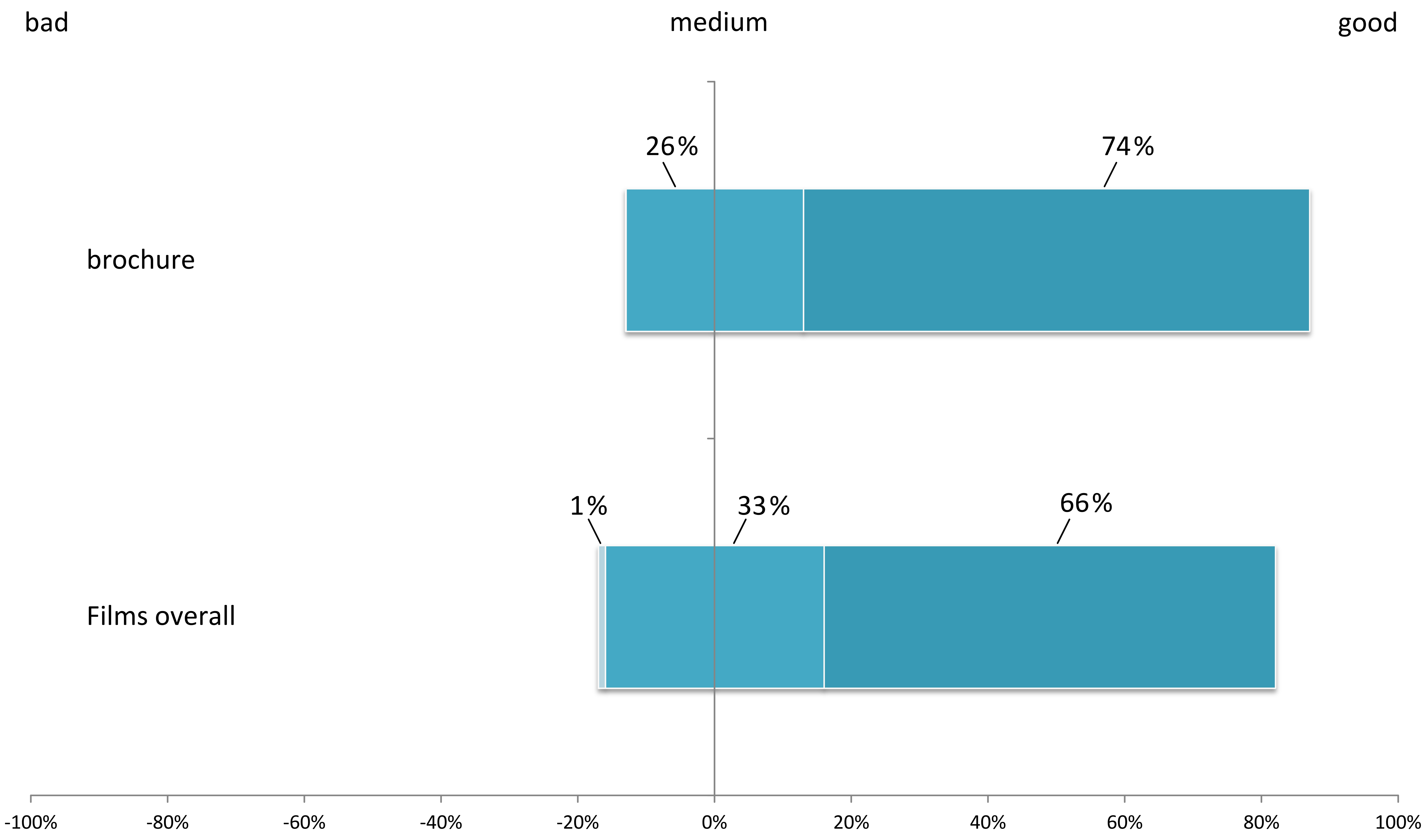

Supplement: Supplementary file 3 — Additional file 3: Figure S3. Evaluation of AHOI-films overall and of the brochure, films n = 209, brochure n = 126. [file 13756_2019_648_MOESM3_ESM.pdf]
